# Language and health-related quality of life outcomes of children early-detected with unilateral and mild bilateral hearing loss

**DOI:** 10.3389/fped.2023.1210282

**Published:** 2023-08-14

**Authors:** Peter Carew, Daisy A. Shepherd, Libby Smith, Qi Rui Soh, Valerie Sung

**Affiliations:** ^1^ Murdoch Children's Research Institute, Royal Children's Hospital, Parkville, VIC, Australia; ^2^ Department of Audiology and Speech Pathology, The University of Melbourne, Parkville, VIC, Australia; ^3^ Department of Paediatrics, The University of Melbourne, Parkville, VIC, Australia; ^4^ Centre for Community Child Health, Royal Children's Hospital, Parkville, VIC, Australia

**Keywords:** unilateral hearing loss, mild bilateral hearing loss, unilateral auditory neuropathy spectrum disorder, early-identified, language outcomes, health-related quality of life

## Abstract

**Introduction:**

We aimed to describe the language and health-related quality of life (HRQoL) outcomes of children early-identified with unilateral or mild bilateral permanent hearing loss. This was a cross-sectional community-based study of children with mild bilateral or unilateral permanent hearing loss (including unilateral auditory neuropathy spectrum disorder (ANSD)), drawn from a population-based databank in Victoria, Australia.

**Methods:**

Enrolment in this databank is independent of early intervention and amplification approaches. Language and caregiver-reported HRQoL outcomes are described by type and degree of loss at three timepoints across child development: at age 2 years (*n* = 255), 5–7 years (*n* = 173) and 9–12 years (*n* = 45).

**Results:**

Across all age groups, average language outcomes were poorer than population normative scores by between a half to two thirds of a standard deviation. Children with mild bilateral hearing loss demonstrated poorer average language outcomes than children with unilateral hearing loss, particularly at younger ages. Children with unilateral ANSD showed language outcomes comparable to their peers with unilateral profound hearing loss. Children had poorer HRQoL psychosocial scores compared to physical scores, without obvious patterns of outcomes linked to degree or type of hearing loss.

**Discussion:**

This study demonstrates children with early-identified unilateral or mild bilateral hearing loss have average language and HRQoL outcomes poorer than population normative expectations from an early age. These outcomes are observed at later ages across childhood. These findings provide a contemporary description of language and quality of life outcomes for children identified but not targeted by universal newborn hearing screening and raise questions of how to provide better support for these populations of children and their families.

## 1. Introduction

Universal newborn hearing screening (UNHS) has had a transformational effect on the development pathways and early life outcomes for children born with congenital hearing loss. It is now common for identification of hearing loss to occur in the first weeks of life (1), facilitating interventions such as amplification and enrolment into early intervention programs earlier than previously routinely possible (2). Earlier identification of hearing loss has led to improved language outcomes, although many children still have language development below expected for their age and cognitive potential (3, 4). The impact of early hearing loss identification on health-related quality of life (HRQoL) is less clear, with some studies documenting improved HRQoL in children whose hearing loss was identified through UNHS compared to without UNHS (5), whilst other studies showed no difference (4).

Many UNHS programs (e.g., in Australia and the United Kingdom) target screening for bilateral hearing losses of moderate or greater degree (6), a cut-point chosen because of evidence that earlier detection of these degrees of losses led to improved language outcomes (7). However, UNHS can and does also identify children with mild degrees of hearing loss and unilateral hearing losses—whether planned (8) or as a “by-product” of targeting bilateral moderate or greater degrees (9). Whether early or later detected, there is growing evidence of harmful effects of mild and unilateral hearing loss on several developmental domains including speech and language (10, 11). Recent amplification data from population-wide government hearing services indicate that children with mild and unilateral hearing loss represent a substantial proportion of the paediatric population presenting for amplification services. Hearing Australia, the national provider of hearing amplification for children in Australia, reports the highest proportion of children first fitted with amplification under 12 months of age have an average hearing loss in the better hearing ear in the range of 0–40 decibels (i.e., a mild bilateral or unilateral loss) (12). Historical age at detection for these children was commonly reported to occur (prior to UNHS) around 4–5 years of age (13), or up to 8 years of age for children with unilateral loss (14). Therefore, UNHS could be viewed to have unintentionally supported the creation of a new group of children with hearing loss—those early detected with mild bilateral or unilateral hearing loss.

This “new” population (15) comes with new challenges from the time that they do not pass their newborn screen. It is recognized that diagnosis—both the duration of time to reach a diagnosis and the certainty of diagnosis—is a different process from significant bilateral losses. The number of appointments required to reach a diagnosis can be much more than for children with larger degrees of loss (16). It is likely that this leads to some stress for those involved, particularly families but also professionals (16, 17). With limited evidence for the outcomes of early-detected children with these types of loss, clinical management of these children is challenging (16, 17). Clinical practice guidelines reflect the uncertainty in outcomes for children with mild and unilateral hearing loss, with references to individual observations, watchful waiting, behavioral verification of hearing levels and needs-based approaches to the decision of if and when to provide amplification (e.g., King (18), Fitzpatrick et al. (19)).

Uncertainty, both in outcomes and management approaches, also exists for children with unilateral auditory neuropathy spectrum disorder (ANSD). This is a rare hearing profile, with estimates suggesting individuals with unilateral ANSD comprise 1%–7% of all ANSD cases (20). However, these children are also detected early and the parental uncertainty regarding appropriate approaches to supporting development of language and communication reported for children with bilateral ANSD (21) may also be a factor for their unilateral ANSD peers. Outside of case reports, published studies including individuals with unilateral ANSD have focused on describing the clinical characteristics of impacted individuals (20, 22) or detail electrophysiological traits and characteristics (23) rather than their developmental outcomes.

This study addresses the gap in literature on the outcomes of children with early-detected mild and unilateral hearing loss. We describe the language and HRQoL outcomes of a contemporary population of children with different degrees of non-target hearing loss (i.e., hearing loss that was not the target for UNHS in Australia) including unilateral ANSD at different ages across child development.

## 2. Methods

### 2.1. Study design and participants

This was a cross-sectional study of Australian children whose degree of permanent hearing loss at diagnosis was either of mild degree in at least the better ear (grouped as mild bilateral), or unilateral of any degree (mild, moderate, severe or profound). This group represents the group of children whose hearing was not the target for UNHS (i.e., not bilateral moderate to profound) in Australia. Children with a diagnosis of unilateral ANSD were also included. Outcomes of participants, collected between 2014 and 2023, were drawn from set data-collection points of a databank built to track the developmental outcomes of children born with permanent hearing loss, the Victorian Childhood Hearing Longitudinal Databank (VicCHILD).

VicCHILD is a population-level data repository, open to all children born or living in the state of Victoria, Australia, with any degree and type of permanent hearing loss. Recruitment into VicCHILD is currently still active, and since its inception in 2012 has over 1,200 participant families. Most VicCHILD participants are under one year of age at enrolment. The majority of participants also have access to government-supported hearing amplification and early intervention programs. Data are collected longitudinally via repeated measures across childhood, at enrolment and at key developmental stages: preschool (∼2 years), primary school entry (5–7 years), and primary school exit (9–12 years). Data are collected either via caregiver-report or direct assessment, across domains covering health, physical development, quality of life, language and listening. More details on the VicCHILD methodology are available elsewhere (24). VicCHILD has ethics approval from the Royal Children's Hospital Human Research and Ethics Committee (approval number 31081), with parent/caregivers providing written informed consent.

#### 2.1.1. Recruitment

The primary recruitment mechanism for VicCHILD is via Victoria's UNHS program, the Victorian Infant Hearing Screening Program (VIHSP), which routinely screens 99.5% of babies in the days and weeks after birth and supports families through to the point of definitive diagnosis of hearing loss (25). VIHSP sends a letter about VicCHILD to eligible families whose child has a confirmed hearing loss diagnosis from diagnostic audiology. This letter provides a two-week window for families to opt-out of learning about VicCHILD, after which time VIHSP passes contact details to the VicCHILD research team who contacts eligible families. The VicCHILD research team describes the databank and obtains consent to provide further details—after which time families decide whether to join the databank and provide consent to participate.

### 2.2. Outcome measures

This study reports VicCHILD's language and HRQoL outcomes in 3 different age groups, using normed and standardized measures, as described below. They were collected as part of multiple other outcome measures collected at the 3 different developmental age brackets (further details described elsewhere) (24).

#### 2.2.1. Language measures

##### 2.2.1.1. 2 years: expressive vocabulary

At around age 2 years, VicCHILD families received and completed either a paper-based or online survey. The primary language outcome collected for this age-group is caregiver-reported expressive vocabulary. This was measured by the 100-word checklist from the Sure Start Language Measure (SSLM) (26), designed for expressive vocabulary assessment across ages 16–30 months. To complete this measure, caregivers indicate which words from the provided list their child says. This measure, based upon the MacArthur Bates Communicative Development Inventory: UK Short Form (27), demonstrates high reliability and concurrent validity (26) and is standardized (based on the child's sex and age in months) with a mean expected score of 100, standard deviation of 15.

##### 2.2.1.2. 5–7 years and 9–12 years: expressive and receptive language and receptive vocabulary

At both 5–7 years and 9–12 years timepoints, language outcomes were collected by direct-assessment measures, completed at a location convenient to the family (at home, at the Royal Children's Hospital, or online during the COVID−19 pandemic). For children who underwent the same assessments within the specified age brackets as part of their usual clinical care, families provided permission for these assessment results to be shared with the research team.

###### 2.2.1.2.1. Clinical evaluation of language fundamentals recalling sentences test

The Clinical Evaluation of Language Fundamentals fourth edition (CELF-4, Australian Version) is a normed measure used both clinically and in educational settings to assess receptive and expressive language (28). The Recalling Sentences test is one subscale from the CELF-4, which along with three other subscales is used to calculate a Core Language Score. However, the Recalling Sentences test administered in isolation has been demonstrated in a large Australian population-based study to be a strong predictor of the total CELF Core Language scores (29). Consequently, we used the Recalling Sentences test as a marker of both expressive and receptive language ability. The Recalling Sentences test is standardized for the ages 5–21 years.

The Recalling Sentences test was administered via an iPad, with children repeating an audio-recorded sentence they have heard, verbatim. This method allows assessment without visual cues. Sentence length and difficulty would progress across the test. Responses are scored live by trained research assistants, rated as either “correct” (no errors), “intermediate/uncertain” (two or three errors) or “incorrect” (four or more errors). The Recalling Sentences test ends after 32 sentences, or after three consecutive “incorrect” scores. A raw score is obtained for each child ranging from 0 to 96. From this, conversion to an age-related scaled score occurs (possible values spanning 1 to 18), with a normative data mean of 10 and standard deviation of 3.

###### 2.2.1.2.2. National institute of health toolbox picture vocabulary test

Receptive vocabulary was assessed using an adaptive test, the National Institutes of Health Toolbox Picture Vocabulary Test (NPVT) (30). The NPVT is a validated measure of general vocabulary knowledge for children aged between 3 and 17 years. On an iPad, children see four images and are required to select the image that best/most closely represents the audio recording of a word played to them. Following two practice items, up to 25 test items with a wide range of difficulty are delivered, with adjustment to difficulty made automatically according to the child's performance on the preceding word.

A theta score (similar to a *z*-score) is reported by the application at the conclusion of the test; representing the relative overall performance of the child. The NPVT provides age-adjusted, fully adjusted and unadjusted scale scores (standard scores), as well as a national percentile rank that corresponds to the age-adjusted scale score. VicCHILD calculates the standard score, which is the receptive vocabulary outcome used in this study. Based on Toolbox normative data, all scaled scores can be interpreted to understand individual performance. An age-adjusted scale score around 100 suggests vocabulary ability is at the expected level for the child's age, with scores of 115 suggesting above-average ability. A score of 85 represents below-average vocabulary ability.

#### 2.2.2. Health-related quality of life measures: all age groups

To measure HRQoL, the Pediatric Quality of Life (PedsQL) (31) was used. A generic instrument validated for use in populations with hearing loss, the PedsQL is a standardized measure with 23 items; we used the Generic Core Scale, V4.0 in this study. The tool comprises 23 items across four domains: Physical, Emotional, Social, and School Functioning (31). With a five-point response scale for each item reverse scored and transformed to a 0–100 scale, a score of 100 represents the best possible HRQoL in relation to questions about how much certain tasks or activities were a problem for the child.

In addition to the total score, two summary metrics are also produced from the PedsQL questionnaire: the physical health summary score, and the psychosocial health summary score. The caregiver proxy-report version was used at all ages in this study, a format demonstrated to have reliability and validity in these age groups of interest (32). Caregivers were asked to consider the child over the past one month when answering each item. Caregivers completed the PedsQL at 2 years or around 5–7 years and 9–12 years at the time of the language assessment.

### 2.3. Hearing loss characteristics

The definition of hearing loss for this study reflects that used by VicCHILD (24). The primary source of information on hearing loss at enrolment were UNHS records. At scheduled contact points with participating families, hearing loss records were updated using caregiver-supplied audiology reports.

Degree of hearing loss was classified using decibel ranges used by the national provider of hearing amplification, Hearing Australia (33): mild (21–40 dB), moderate (41–60 dB), severe (61–90 dB) and profound (>90 dB). Participants were recorded as having either a unilateral or bilateral hearing loss based on the presence/absence of hearing loss in the second ear. A diagnostic report stating the presence of unilateral ANSD, with normal hearing in the second ear, was used to identify our unilateral ANSD sample for this study.

Type of hearing loss for VicCHILD is not restricted to sensorineural losses. Due to this, our study sample included a small number of children identified with permanent conductive and mixed hearing losses. Children identified with unilateral aural atresia were excluded from this study as their outcomes are reported elsewhere.

### 2.4. Other participant characteristics

Participant characteristics were collected at enrolment and updated at each data collection point. The participant characteristics included in this study's analyses were demographic characteristics (sex, age at assessment, socioeconomic disadvantage, household income, household primary language, maternal education level) and health-related characteristics (number of comorbidities, gestational age, non-verbal IQ and whether an individual was admitted to NICU). From 2020 onwards, caregivers were asked to report on their child's additional health needs or medical diagnoses.

### 2.5. Study sample selection

This study included all VicCHILD participants identified to have a hearing loss diagnosis satisfying the criteria of mild bilateral hearing loss in the better ear, or a unilateral hearing loss of any degree, identified by VIHSP, born between 2005 and 2020, with data collected between December 2014 and March 2023. For each age group, children were included in the study sample if they had at least one outcome (language or HRQoL) measured at that data collection point. Hearing and demographic data were collated from data recorded at the first two collection points (enrolment and age 2 years). Updated service and device use data were also obtained at each subsequent collection point (age 5–7 years and 9–12 years).

Three study samples were formed corresponding to the three timepoints across child development, at age 2 years (early life), 5–7 years (entry to primary school) and 9–12 years (transition to secondary school), respectively. Due to the longitudinal nature of the VicCHILD databank, data from some participants were included across multiple age groups and therefore the three samples were not completely independent.

### 2.6. Statistical analysis

For each of the three age groups, key hearing-related, demographic and health-related characteristics were summarized. Continuous measures were reported as means and standard deviations (SD) or medians and interquartile range limits (IQR) depending on their distribution, with categorical characteristics reported as frequencies and proportions. The number of participants common to multiple age groups were quantified and reported.

Outcome measures were reported for all individuals, and then further stratified by degree of hearing loss. For each age group, the mean language measures (i.e., SSLM score, CELF recalling sentences, NPVT) were reported, alongside the SD and associated 95% confidence interval (CI). Due to the skewed nature of the PedsQL measure, the median PedsQL score and IQR were reported, alongside an estimated 95% CI using the Binomial distribution. When stratified by degree of hearing loss, the older age group (9–12 years) had small sample sizes and therefore the CI was not estimated due to low precision. We considered mean scores to represent below average performance if scores were greater than 1 standard deviation below the normative mean, with above average performance represented by scores greater than 1 standard deviation above the normative mean.

All analyses were conducted in R version 4.1.2 (34) using complete case analysis.

## 3. Results

### 3.1. Participant characteristics

Data in this study represent 473 individual records of child outcomes, spread across three timepoints: 2 years (*n* = 255), 5–7 years (*n* = 173) and 9–12 years (*n* = 45). Data from 8 participants were included in all age groups; 79 participants' data were included in both the 2 year and 5–7 years age groups, and 34 participants' data were included in the two older age groups.

Table 1 describes the participant characteristics. Sex proportions across the three timepoints varied somewhat, with 40%–44% of participants at 2 years and 5–7 years reported female, and 53% female at 9–12 years. On average, across all ages, participants lived in areas of slightly less socio-economic disadvantage compared to the Australian population norm (mean Socio-Economic Indexes for Areas (SEIFA) scores of 1,004, 1,007 and 1,023 in increasing age group order, where a higher number represents less disadvantage, compared to normative score of 1,000). Over 80% of participants at all timepoints had reported maternal education completion being at least year 12 (completed high school), and most participants lived in households with high levels of reported income. Participants whose data were collected at the youngest timepoint (2 years) reported the highest proportion of languages used in the home being other than/additional to English. Participants were predominantly well babies, with mean gestational ages reflective of full term pregnancies and more than 80% of births not requiring admission to a neonatal intensive care unit.

**Table 1:** Characteristics of the three study samples.

		Age 2 years		Age 5–7 years		Age 9–12 years	
		*N* = 255		*N* = 173		*N* = 45	
		Missing^a^, *n* (%)		Missing^a^, *n* (%)		Missing^a^ *n* (%)	
Hearing-related characteristics							
Age at detection/diagnosis (months)—median [IQR]		21 (8.24)	1.20 [1.20, 1.20]	9 (5.20)	1.20 (1.20, 2.40)	3 (6.67)	1.50 (1.11, 2.40)
Hearing loss severity—*n* (%)		0 (0)		0 (0)		0 (0)	
Bilateral:	Mild		93 (36.47)		63 (36.42)		12 (26.67)
Unilateral:	Mild		18 (7.06)		14 (8.09)		5 (11.11)
	Moderate		34 (13.33)		25 (14.45)		6 (13.33)
	Severe		35 (13.73)		21 (12.14)		8 (17.78)
	Profound		35 (13.73)		34 (19.65)		12 (26.67)
ANSD (unilateral)			40 (15.69)		16 (9.25)		2 (4.44)
Type of hearing loss—*n* (%)		0 (0)		1 (0.62)		0 (0)	
SNHL			199 (78.04)		144 (83.72)		28 (84.44)
Auditory neuropathy			40 (16.59)		16 (9.30)		2 (4.44)
Mixed HL			8 (3.14)		5 (2.91)		1 (2.22)
Conductive HL			5 (1.96)		7 (4.07)		4 (8.89)
Not available/applicable			3 (1.18)		0 (0)		0 (0)
Amplification status at time of survey—*n* (%)		44 (17.25)		25 (14.45)		4 (8.89)	
No device			117 (55.45)		67 (45.27)		24 (58.54)
Hearing aid(s) only			89 (42.18)		76 (51.35)		17 (41.46)
CI (unilateral or bilateral) only			4 (1.90)		0 (0)		0 (0)
Hearing aid and CI			1 (0.47)		5 (3.38)		0 (0)
Frequency of device use at time of survey: *n* = 94/81/17		3 (3.19)		31 (38.27)		4 (23.53)	
<4 h			24 (26.37)		1 (2.00)		0 (5.26)
4–8 h			39 (42.86)		17 (34.00)		7 (53.85)
>8 h			28 (30.77)		32 (64.00)		6 (46.15)
Age first device fitted (months)^b^—median (IQR)		129 (50.59)	6.00 (3.00, 12.75)	92 (53.18)	18.00 (6.00, 46.00)	22 (48.89)	24.00 (9.00, 54.50)
Enrolled in early intervention services							
Ever—*n* (%)		36 (14.88)	107 (48.86)	72 (41.62)	51 (50.50)	DNC	DNC
Age at enrolment—median [IQR]		160 (62.75)	8.00 [5.00, 13.50]	DNC	DNC	DNC	DNC
Demographic characteristics							
Age at language assessment (years)—mean (SD)		58 (22.75)	2.14 (0.16)	19 (10.98)	6.90 (0.78)	8 (17.78)	11.27 (0.94)
Age at PedsQL completion (years)—mean (SD)		2 (0.08)	2.31 (0.26)	36 (20.81)	6.72 (0.79)	3 (6.67)	11.21 (0.99)
Sex of child: Female—*n* (%)		0 (0)	103 (40.39)	0 (0)	76 (43.93)	0 (0)	24 (53.33)
Socioeconomic disadvantage (SEIFA)—mean (SD)		0 (0)	1,004.72 (63.35)	0 (0)	1,007.17 (69.05)	0 (0)	1,023.93 (68.34)
Household income—*n* (%)		28 (9.80)		23 (13.29)		4 (8.89)	
<$31,199			19 (8.37)		10 (6.67)		3 (7.32)
$31,199—$51,999			21 (9.25)		18 (12.00)		3 (7.32)
$52,000—$103,999			94 (41.41)		75 (50.00)		20 (48.78)
>$104,000			93 (40.97)		47 (31.33)		15 (36.59)
Household primary language—*n* (%)		33 (12.94)		42 (24.28)		29 (64.44)	
English only			113 (50.90)		75 (57.25)		11 (68.75)
Bilingual/multilingual (English + other)			75 (33.78)		43 (32.82)		2 (12.50)
Other language(s) only			34 (15.32)		13 (9.92)		3 (18.75)
Maternal education—*n* (%)		19 (7.45)		28 (16.18)		21 (53.33)	
Year 10 or less			24 (10.17)		16 (11.03)		3 (12.50)
Year 11			12 (5.08)		6 (4.14)		1 (4.17)
Year 12			61 (25.85)		51 (35.17)		11 (45.83)
Tertiary or postgraduate			139 (58.90)		72 (49.66)		9 (37.50)
Health-related characteristics							
Number of comorbidities—*n* (%)		122 (47.84)		67 (38.73)		15 (33.33)	
None			54 (40.60)		35 (33.02)		8 (26.67)
1			45 (33.83)		29 (27.36)		7 (23.33)
2			22 (16.54)		22 (20.75)		8 (26.67)
3 or more			12 (9.02)		20 (18.87)		7 (23.33)
NICU admissions: yes—*n* (%)		8 (3.14)	48 (19.43)	9 (5.20)	26 (15.85)	1 (2.22)	7 (15.91)
Gestational age—mean (SD)		4 (1.57)	38.46 (2.38)	0 (0)	38.82 (2.43)	1 (2.22)	39.02 (2.44)
Non-verbal IQ—mean (SD)		*DNC*	*DNC*	17 (9.83)	102.51 (18.39)	2 (4.44)	100.00 (18.22)

^a^ Relative to sample size for each age point unless specified in the left-hand column.

^b^ Note the high level of missing information due to a large proportion of individuals most likely not ever having a device. However, this information was not collected via our data collection tool so we are unable to quantify this.

*DNC* corresponds to a cell in which that data/information was not collected at that time point.

Consistent with expectations of UNHS, children were diagnosed with hearing loss early in life with median age at detection ranging from 1.2 to 1.5 months across all three timepoints (Table 1). A quarter to a third of participants at all timepoints were diagnosed with a mild bilateral hearing loss; most children with unilateral hearing loss had a profound degree of loss. Participants with unilateral ANSD represented 15% of our sample at 2 years. The majority of participants had sensorineural hearing loss, with smaller proportions with mixed and permanent conductive losses, reflecting the source of the sample—from a population-based databank inclusive of all children with permanent hearing loss of any degree or type. Around 60%–75% of participants were reported to have one or more additional health need or medical diagnosis in addition to hearing loss.

The majority of participants had no hearing device fitted at the time of assessment (2 years, 55%; 9–12 years, 58%) or had hearing aid only (5–7 years, 51%). For those fitted with hearing device(s), the median age of first fitting was lowest in the younger data collection timepoints, with a median age of 6 months for participants at age 2 years (IQR: 3.0, 12.8 months) (Table 1). We observed greater proportions of hearing device use at timepoints when participants were older. Half (50%) of participants had never engaged with an early intervention program at the time data were collected at 2 years and 5–7 years. At the two timepoints where non-verbal IQ testing was possible, mean IQ scores reflected population normative scores (5–7 years, mean IQ 102 (SD 18); 9–12 years, mean IQ 100 (SD 18)).

### 3.2. Language

#### 3.2.1. Early life (2yo)

When considered as a single group, children at age 2 years with unilateral or mild bilateral loss in our sample demonstrated, on average, caregiver-reported expressive vocabulary approximately two thirds of a standard deviation below population normative scores (*n* = 197, mean 90.5, 95% CI: 88.22, 92.74) (Table 2).

**Table 2:** Summary of language and vocabulary scores for each age group.

Age 2 years		Expressive vocabulary (SSLM)							
		*n*	Mean	SD	95% CI				
Overall		197	90.48	16.09	(88.22, 92.74)				
By hearing loss									
Bilateral:	Mild	69	88.39	16.88	(84.34, 92.45)				
Unilateral:	Mild	11	95.73	18.75	(83.13, 108.32)				
	Moderate	28	86.32	15.47	(80.32, 92.32)				
	Severe	30	94.90	16.05	(88.91, 100.89)				
	Profound	26	91.85	15.61	(85.54, 98.15)				
ANSD (unilateral)		33	91.55	13.76	(86.67, 96.42)				
Age 5–7 years		CELF Recalling sentences				NPVT			
		*n*	Mean	SD	95% CI	*n*	Mean	SD	95% CI
Overall		146	8.05	3.76	(7.44, 8.67)	144	90.85	21.28	(87.34, 94.36)
By hearing loss									
Bilateral:	Mild	53	6.92	3.97	(5.83, 8.02)	52	82.32	25.54	(75.21, 89.44)
Unilateral:	Mild	10	7.60	4.58	(4.33, 10.87)	10	88.73	22.91	(72.35, 105.12)
	Moderate	22	9.18	3.59	(7.59, 10.78)	20	94.84	12.84	(88.83, 100.85)
	Severe	17	9.59	3.74	(7.66, 11.51)	16	96.15	17.85	(86.64, 105.67)
	Profound	30	8.23	2.88	(7.16, 9.31)	31	95.67	17.52	(89.24, 102.1)
ANSD (unilateral)		14	8.64	3.48	(6.63, 10.65)	15	100.87	14.20	(93.01, 108.74)
Age 9–12 years		CELF Recalling sentences				NPVT			
		*n*	Mean	SD	95% CI	*n*	Mean	SD	95% CI
Overall		37	8.49	3.49	(7.32, 9.65)	36	98.42	17.88	(92.37, 104.46)
By hearing loss									
Bilateral:	Mild	9	9.22	3.19	–	8	104.93	12.52	–
Unilateral:	Mild	5	7.60	5.41	–	5	89.49	34.46	–
	Moderate	5	8.60	1.82	–	5	96.54	9.12	–
	Severe	7	8.29	3.68	–	7	100.92	14.29	–
	Profound	9	7.89	3.72	–	9	99.95	17.52	–
ANSD (unilateral)		2	10.5	3.54	–	2	83.69	8.30	–

Children with mild bilateral and moderate unilateral losses demonstrated the poorest expressive vocabulary, with mean scores approaching a full standard deviation below population normative scores at this young age (mean 88.4, 95% CI: 84.3–92.5, and 86.3, 95% CI: 80.3, 92.3, respectively).

**Figure 1:**
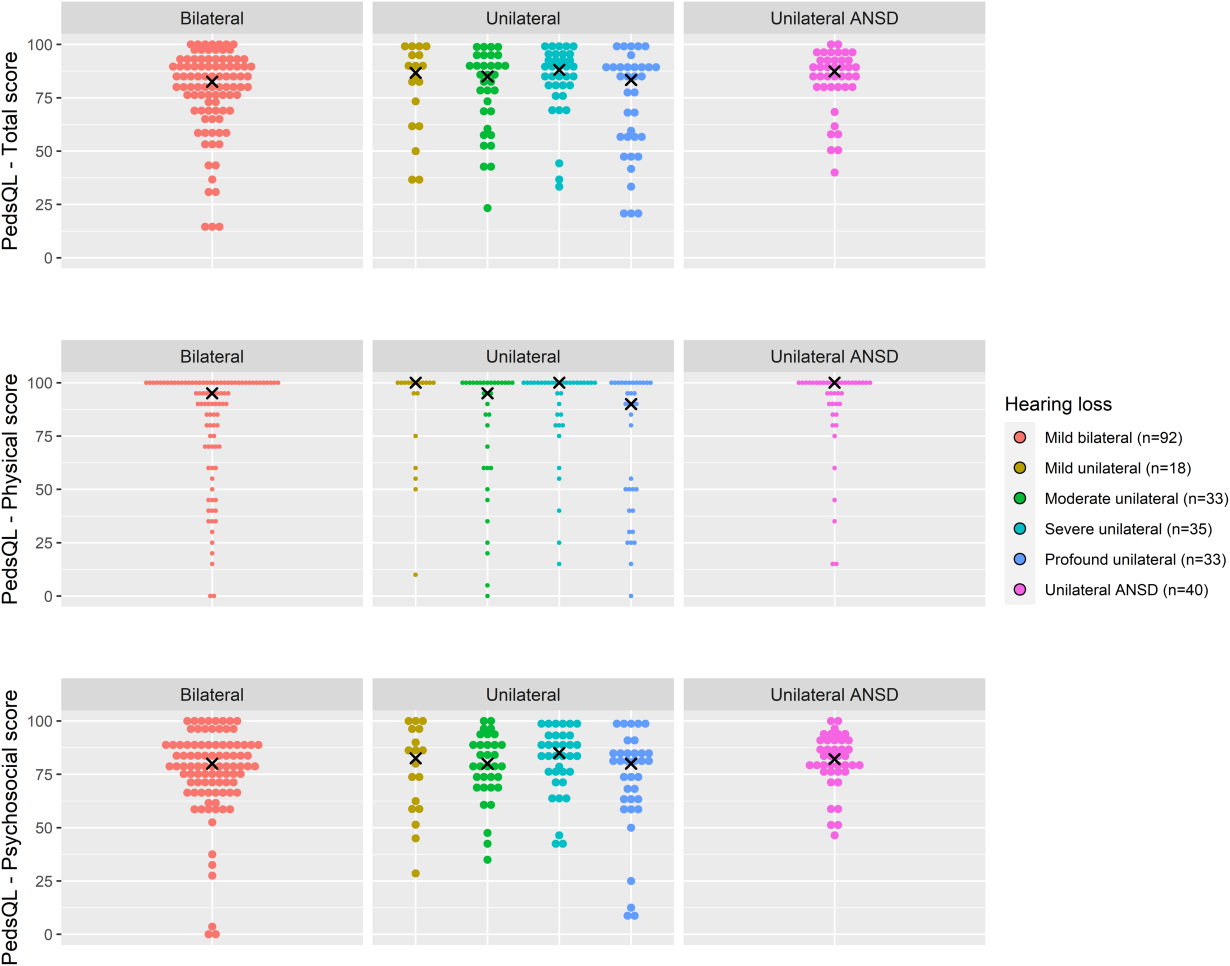
Expressive vocabulary (standardised SSLM scores) for age 2 years across hearing loss groups.

When considering unilateral sensorineural losses, we observed little difference in expressive language across children with mild, severe and profound losses on average, with mean vocabulary scores ranging from one third to two thirds of a standard deviation poorer than population normative scores (Figure 1), although not substantially lower comparatively to the population scores (e.g., 95% CIs presented in Table 2).

Children with unilateral ANSD demonstrated expressive vocabulary scores around two thirds of a standard deviation below population normative scores (mean 91.6, 95% CI: 86.7–96.4), a comparable mean outcome to those with profound unilateral loss (mean 91.9, 95% CI: 85.5–98.2) (Figure 1; Table 2).

#### 3.2.2. Entry to primary school (5–7yo)

Language outcomes at this age group were, in general, poorer than population normative scores. Used as a marker of expressive and receptive language, scores on the CELF Recalling Sentences subscale suggest that when considered as a single group, children in the early primary school years with unilateral or mild bilateral hearing loss in our sample were scoring approximately two thirds of a standard deviation, on average, poorer than population normative scores (*n* = 146, mean 8.1, 95% CI: 7.4–8.7) (Table 2).

**Figure 2:**
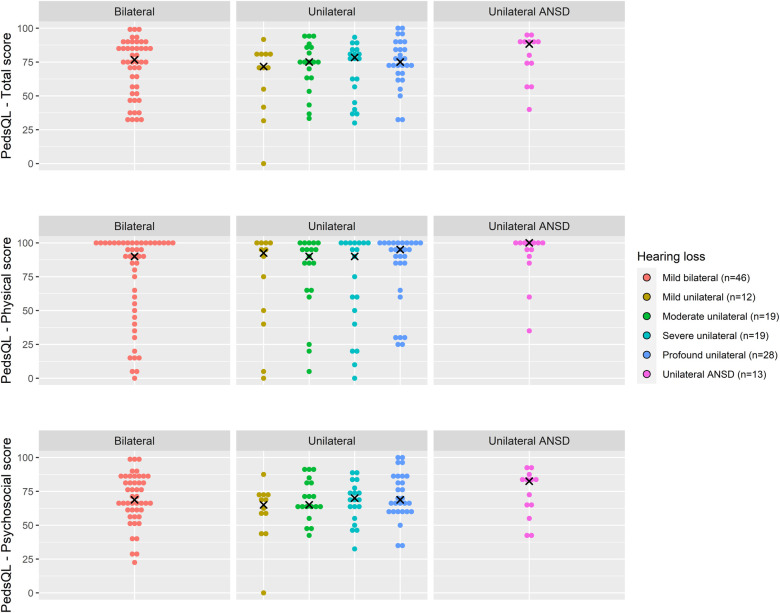
Language (CELF recalling sentences) and vocabulary (NPVT) scores for age 5–7 years across hearing loss groups.

At this age point, children with mild bilateral hearing loss were, on average, one standard deviation below population normative scores (mean 6.9, 95% CI: 5.8–8.0), the poorest average performance of any hearing loss group (Figure 2; Table 2). Across unilateral sensorineural losses, we observed mean language performance within one standard deviation of population normative scores, and those with moderate, severe or profound losses having some scores approaching and exceeding the expected standardized score of 10 (Figure 2; Table 2).

Children with unilateral ANSD demonstrated a range of language performances roughly similar to children with profound unilateral losses (mean 8.6, 95% CI: 6.6–10.7, and mean 8.2, 95% CI: 7.2–9.3, respectively) (Table 2).

Of the 144 children on whom receptive vocabulary assessment had been conducted, we observed the greatest variability in performance for children with mild bilateral hearing loss in our sample (SD 25.5 points, Figure 2). On average, these children had receptive vocabulary scores in the below average range (mean 82.3, 95% CI: 75.2–89.4) (Table 2).

For children with unilateral sensorineural loss, their mean receptive vocabulary scores were closer to the expected score of 100, but still slightly poorer than population normative scores (Figure 2; Table 2) with a smaller spread of scores than observed for mild bilateral losses. Of the 15 children with unilateral ANSD, receptive vocabulary performance was, in general, within the expected performance range (85 to 115) (Figure 2).

#### 3.2.3. Transition to secondary school (9–12yo)

**Figure 3:**
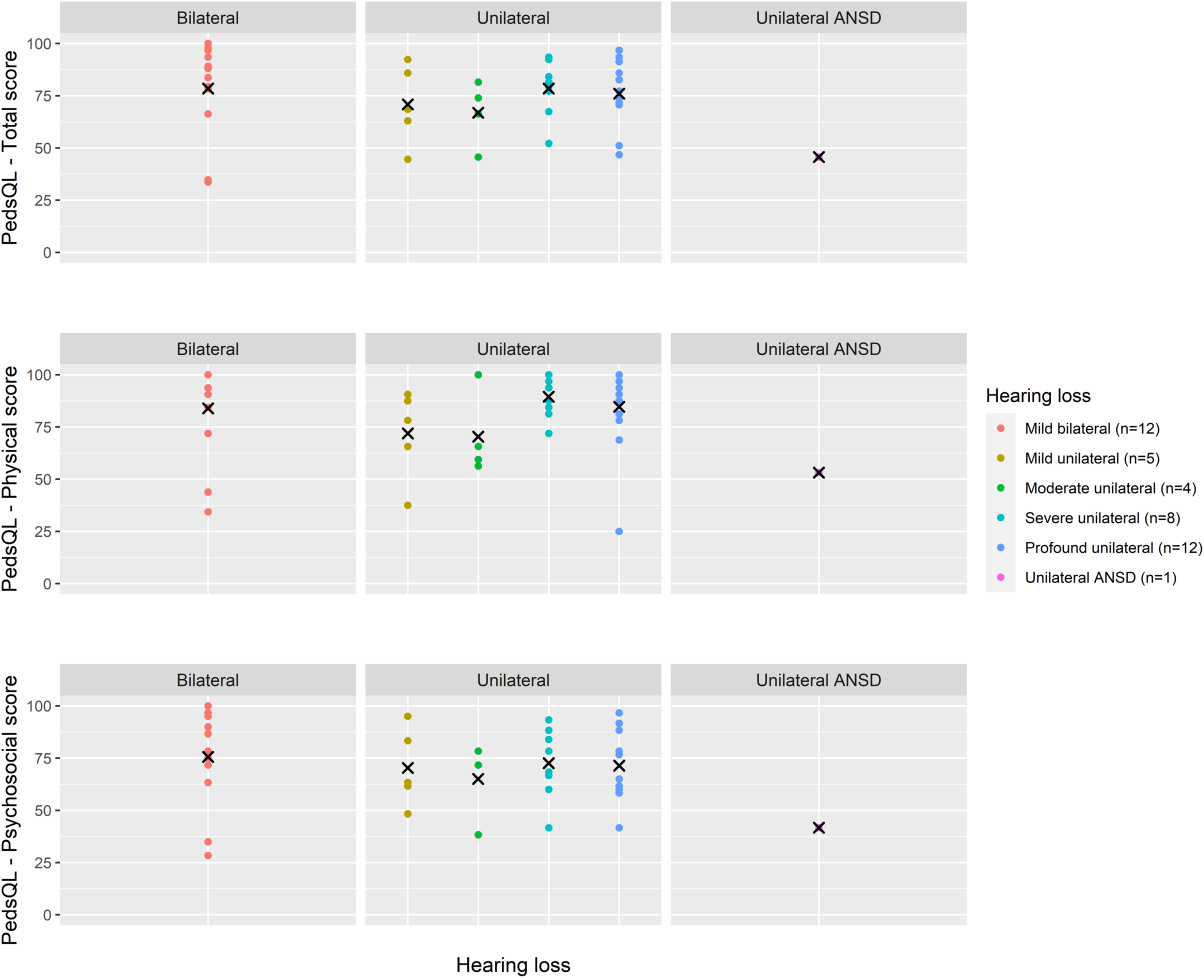
Language (CELF recalling sentences) and vocabulary (NPVT) scores for age 9–12 years across hearing loss groups.

Similar to the pattern of performance seen at the entry to primary school age group, more individual performance variation was observed for expressive and receptive language outcomes in the transition to secondary school group as opposed to patterns of performance for receptive vocabulary outcomes—where mean scores approximated population normative scores (Figure 3; Table 2).

Due to small sample sizes within discrete degrees of hearing loss in this age group, aggregate results were described. Overall, mean expressive and receptive language outcomes at this age in our sample were around half a standard deviation poorer than population normative scores (*n* = 37, mean 8.5, SD 3.5) (Table 2). Mean receptive vocabulary scores were close to population normative scores (*n* = 36, mean 98.4, SD 17.9).

### 3.3. Health-related quality of life

Due to skewed distribution of HRQoL scores, median scores were presented. Overall, physical PedsQL scores in all age groups and for all degrees of loss were higher than psychosocial PedsQL scores (Table 3). Psychosocial PedsQL scores had a wider distribution in individual performance than physical PedsQL scores; this was particularly noticeable at our early life (2 years) and entry to primary school (5–7 years) timepoints, and was observed for all degrees of loss.

**Table 3:** Summary of health-related quality of life outcomes for each age group.

		*n*	PedsQL Total score			PedsQL Physical score			PedsQL Psychosocial score		
			Median	IQR	95% CI	Median	IQR	95% CI	Median	IQR	95% CI
Age 2 years											
Overall		253	85.00	[69.52, 92.86]	(83.33, 86.67)	95.00	[70.00, 100.00]	(95.00, 100.00)	80.00	[70.00, 90.00]	(78.57, 82.50)
By hearing loss											
Bilateral:	Mild	92	82.50	[70.00, 91.41]	(79.27, 86.67)	95.00	[70.00, 100.00]	(90.00, 100.00)	80.00	[69.46, 89.29]	(75.00, 82.50)
Unilateral:	Mild	18	86.67	[65.83, 95.83]	(63.33, 96.67)	100.00	[80.00, 100.00]	(75.00, 100.0)	82.50	[60.63, 93.75]	(60.00, 95.00)
	Moderate	33	85.00	[68.33, 91.67]	(73.33, 90.48)	95.00	[60.00, 100.00]	(70.00, 100.00)	80.00	[70.00, 90.00]	(72.50, 87.50)
	Severe	35	88.1	[80.36, 94.17]	(81.67, 92.86)	100.00	[82.50, 100.00]	(90.00, 100.00)	85.00	[75.00, 92.50]	(77.50, 89.29)
	Profound	35	83.33	[55.00, 89.40]	(58.33, 88.33)	90.00	[45.00, 100.00]	(50.00, 100.00)	80.00	[63.39, 85.36]	(67.50, 85.00)
ANSD (unilateral)		40	87.38	[80.00, 93.33]	(83.33, 90.48)	100.00	[88.75, 100.00]	(90.00, 100.00)	82.14	[76.88, 90.00]	(78.57, 87.50)
Age 5–7 years											
Overall		137	76.67	[56.67, 86.67]	(73.33, 80.00)	95.00	[60.00, 100.00]	(90.00, 95.00)	70.00	[60.00, 82.50]	(65.00, 72.50)
By hearing loss											
Bilateral:	Mild	46	76.67	[54.17, 86.67]	(65.00, 85.00)	90.00	[51.25, 100.00]	(75.00, 100.00)	68.75	[60.00, 84.38]	(65.00, 80.00)
Unilateral:	Mild	12	71.67	[51.67, 80.42]	(41.67, 81.67)	92.50	[47.50, 100.00]	(40.00, 100.00)	65.00	[54.38, 72.50]	(45.00, 72.50)
	Moderate	19	75.00	[63.33, 85.83]	(61.67, 86.67)	90.00	[65.00, 97.50]	(65.00, 100.00)	65.00	[62.50, 81.25]	(62.50, 82.50)
	Severe	19	78.33	[50.83, 82.50]	(45.00, 83.33)	90.00	[45.00, 100.00]	(40.00, 100.00)	70.00	[58.75, 76.25]	(55.00, 72.50)
	Profound	28	75.00	[66.67, 85.83]	(71.67, 85.00)	95.00	[80.00, 100.00]	(85.00, 100.00)	68.75	[60.00, 85.00]	(60.00, 82.50)
ANSD (unilateral)		13	88.33	[73.33, 90.00]	(56.67, 91.67)	100.00	[90.00, 100.00]	(85.00, 100.00)	82.50	[65.00, 85.00]	(55.00, 87.50)
Age 9–12 years											
Overall		42	78.80	[66.58, 88.86]	[70.65, 84.09)	89.06	[71.88, 96.88]	(81.25, 93.75)	73.33	[61.67, 86.67]	(63.33, 78.33)
By hearing loss											
Bilateral:	Mild	12	85.57	[75.27, 94.29]	–	93.75	[81.25, 100.00]	–	82.50	[69.58, 91.25]	–
Unilateral:	Mild	5	68.48	[63.04, 85.87]	–	78.13	[65.63, 87.50]	–	63.33	[61.67, 83.33]	–
	Moderate	4	70.11	[61.14, 75.82]	–	62.50	[58.59, 74.22]	–	71.67	[63.33, 73.33]	–
	Severe	8	80.43	[74.73, 86.17]	–	90.63	[83.59, 97.66]	–	73.33	[65.00, 85.03]	–
	Profound	12	75.54	[70.65, 87.23]	–	92.19	[80.47, 96.88]	–	70.83	[61.25, 80.83]	–
ANSD (unilateral)		1	45.65	–	–	53.13	–	–	41.67	–	–

The cluster of high HRQoL scores seen at age 2 years was not so pronounced at 5–7 years and this was reflected in the shift in median psychosocial PedsQL scores (2 years psychosocial PedsQL median 80.0, IQR 70 to 90, 5–7 years psychosocial PedsQL median 70.0, IQR 60 to 82.5) (Table 3). Of note, the highest median psychosocial PedsQL score at age 5–7 years was seen in children with unilateral ANSD (median 82.5, IQR 65 to 85).

**Figure 4:**
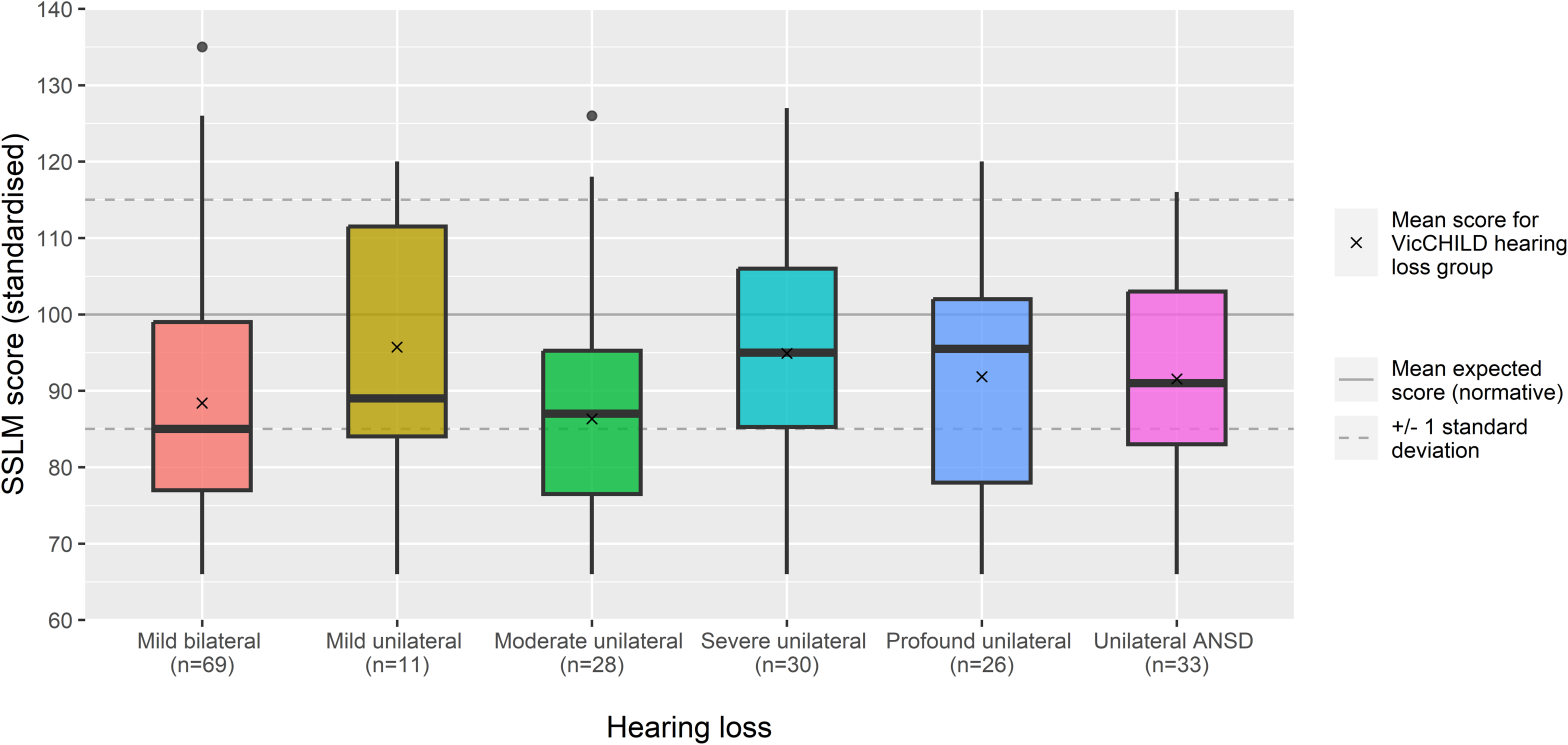
Health-related quality of life (PedsQL 4.0) scores for age 2 years across hearing loss groups.

**Figure 5:**
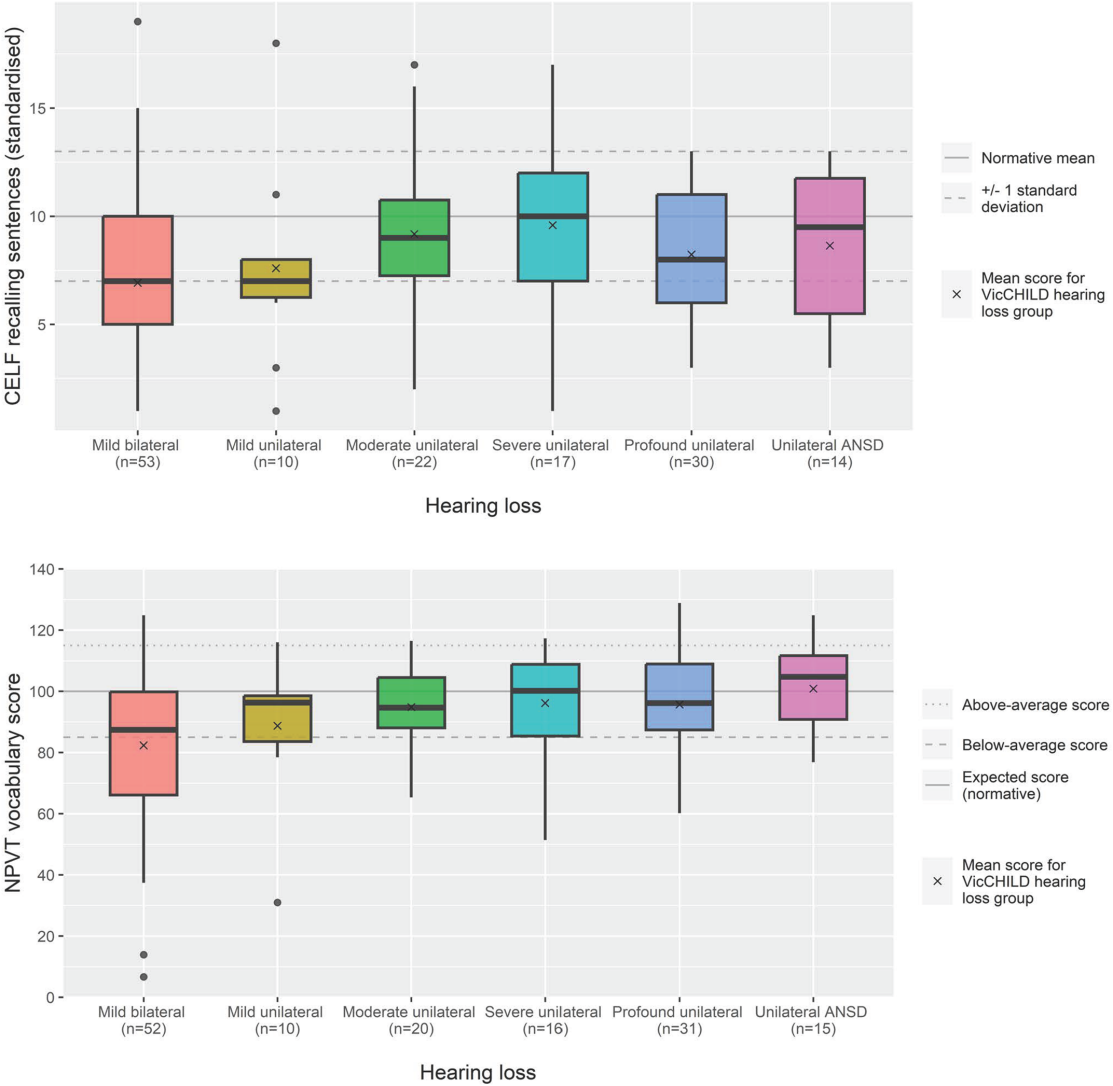
Health-related quality of life (PedsQL 4.0) scores for age 5–7 years across hearing loss groups.

**Figure 6:**
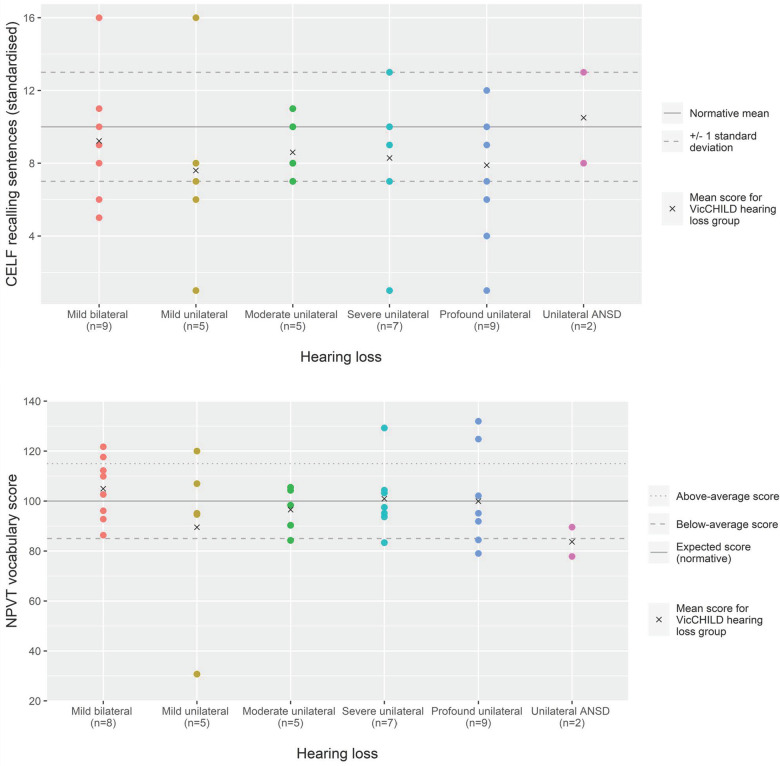
Health-related quality of life (PedsQL 4.0) scores for age 9–12 years across hearing loss groups.

Total PedsQL scores, comprising physical and psychosocial scales, were generally similar across degrees of loss and at all age groups (Figures 4–6).

## 4. Discussion

### 4.1. Principal findings

This study describes language and HRQoL outcomes at multiple age timepoints in a large sample of children across childhood, all of whom had early identified hearing losses not targeted by UNHS in Australia—mild bilateral and unilateral losses.

#### 4.1.1. Language

Across all age groups, overall language outcomes were on average a half to two thirds of a standard deviation poorer than population normative scores.

Children with mild bilateral hearing loss tended to demonstrate poorer language outcomes than those with unilateral loss or unilateral ANSD. This pattern of outcomes was particularly evident at the early life (2 years) and entry to primary school (5–7 years) timepoints.

For children with unilateral hearing loss, receptive vocabulary performance at entry to primary school appeared to be approximating population normative levels. However, receptive and expressive language outcome results tended to be poorer than population normative scores.

Children with unilateral ANSD, across early life and entry to primary school timepoints, demonstrated language performance comparable to children with unilateral profound sensorineural hearing loss. Average language outcome scores were around two thirds of a standard deviation poorer than population normative scores, with similar distributions of performance observed.

Interpreted cautiously due to low participant numbers, children at the transition to secondary school (9–12 year) timepoint were either in general at or within two thirds of a standard deviation below the population normative levels irrespective of degree or type of hearing loss.

#### 4.1.2. Health related quality of life

Across all age groups, children had poorer psychosocial HRQoL scores compared to physical HRQoL scores. Distribution of individual scores appeared to follow the same pattern across all ages and degrees of loss, with most HRQoL scores within the upper quartile scores suggesting many of these children experience high quality of life.

### 4.2. Strengths of the study

A strength of this study is the population-level databank that was the source of participating children. Through this databank we were able to confirm method of hearing loss identification (all detected via UNHS activities) and access outcomes on standardized measures. By using all available timepoints we have been able to maximize the number of results to report outcomes from a large group of children with non-target losses (including unilateral ANSD which has very sparse reporting of language outcomes) detected as by-products of UNHS activities. Our study also provides a description of outcomes at multiple timepoints across child development. This has resulted in a study of the contemporary population that reflects current detection trends (early) and availability of intervention—something that is to our knowledge not available in the extant literature.

Through our recruitment source, we have optimized the reported levels of diversity in participant characteristics that are comparable to the general population—such as levels of socio-economic disadvantage that reflect the expected levels in the Australian population. When compared to clinical samples of children with the same hearing diagnoses, we believe our results are representative of the wider population by documenting varied decisions taken by families around intervention and use of amplification.

### 4.3. Limitations

In reporting descriptive outcomes of children detected with unilateral or mild bilateral hearing loss under contemporary conditions, whilst we have achieved a large sample at 473 data points, we have not explored any causal relationships between degree and type of hearing loss and outcomes for these children. Our study design—drawing on available outcomes from the first decade of an established and growing databank that serves as a repository of outcomes—meant that we cannot yet comment on trajectories of performance across child development, but rather describe age-groups independently. We were also limited to using responses from those families who actively participate in the databank activities but note there were no significant differences in the characteristics of participant responders (Table 1) vs. non-responders (Supplementary Table S1).

The nature of the population databank—where different individual measures need to be as short as possible to reduce participant burden and encourage participant retention over time—precludes the ability to include outcome measures that may have been more sensitive to discrete groups of children with hearing loss. For example, whilst the PedsQL is validated for use in populations with chronic health conditions (32) and has been used in prior studies involving children with hearing loss (35, 36), it may not be as sensitive an instrument as alternate instruments such as the HEAR-QL, in demonstrating potentially more nuanced challenges faced by children with unilateral and mild bilateral hearing loss (37). Moreover, it is not unusual for large databanks that span many years to be challenged by missing data. For example, data about additional health needs or medical diagnoses were collected only after 2020 with a high proportion of missing data for this variable. The reported rate of additional health needs in our sample is higher than that reported in the existing literature (38). This may be because participant families reported against a more comprehensive list of medical diagnoses as compared with previous studies. The higher than expected proportion of children with additional health needs or medical diagnoses could theoretically affect the outcomes measured; however, we do not have complete data for this variable, and we suspect many of our families may have reported on medical diagnoses unrelated to the child's hearing or vocabulary outcomes.

Whilst the number of children we have included in this study is large in relation to many other studies of unilateral and mild bilateral hearing loss, we occasionally interpreted all children's results in one combined group of “minimal” non-target hearing losses. This raises the critique of analyzing outcomes for two different types of hearing loss as one group. It is important to note that children with these hearing loss types are actually heterogenous groups that instead share some common challenges of hearing loss, such as uncertainties in early clinical management and possibly inconsistent early hearing device use (17), and low access to/engagement in early intervention services as demonstrated by our data (less than 50% ever accessed early intervention services). It is possible that the reasons for these challenges may differ between mild bilateral and unilateral losses (39), and it would be preferable to uniformly report their outcomes as discrete groups.

### 4.4. Interpretation in light of other studies

Our study is, in effect, an audit reporting language and HRQoL outcomes in a large group of children born with mild bilateral or unilateral hearing loss. Participants represent the diversity seen in the community with regards to decisions on amplification and intervention that is harder to achieve in clinical samples. Due to the duration of UNHS in Victoria and the size of the databank where participants were drawn from, our study is able to describe outcomes across a larger sample of universally early-identified children than we are aware has been performed prior. Therefore, we believe this study represents a valuable addition to the literature on language and HRQoL outcomes that are seen in the contemporary hearing detection landscape where early detection is common and management decisions vary.

#### 4.4.1. Mild bilateral and unilateral hearing loss

Our early life timepoint results demonstrated poorer expressive vocabulary performance than population norms, aligning with other reports of early life impact of unilateral hearing loss. In a UNHS detected sample with a median age 9.4 months, children with unilateral hearing loss were shown to demonstrate delays in auditory behaviour and preverbal vocalizations when compared to age-matched peers from the same population with normal hearing (40). However, not all reports agree, with another report of early detected children with unilateral and mild bilateral hearing losses showing language development meeting expectations through to four years of age (41). Of note, less than half of our sample of children engaged with early intervention services, possibly a reflection of the availability of these services to this non-target group of children, or low engagement due to perceptions these children may not require such services. With early detection of mild and unilateral losses now routine, it is important to reflect on whether this group of children have access to and are adequately supported to enroll in early intervention services.

Our entry to primary school timepoint demonstrated differences in performance across language and vocabulary outcome measures. This may have to do with task complexity, with our measure of receptive and expressive language (CELF Recalling Sentences) appearing more robust at highlighting performance differences compared to our receptive vocabulary (NPVT) assessment task. With receptive and expressive language requiring skills in morphological and phonological awareness, semantics, syntax and working memory, it may not be surprising that our children with mild bilateral or unilateral loss showed more variation in performance on this task—and lower achievement levels—when compared to the receptive vocabulary task that relies on semantics alone. Challenges in discrete areas of language may be supported by other findings, such as Nassrallah et al. (42) who reported findings of a descriptive study of children aged 5–9 years of age. They reported poorer than expected phonological processing skills, with 46% of children with mild bilateral or unilateral loss more than one standard deviation poorer than the expected level on a phonological memory task.

The results of this study support the conclusion that children with mild bilateral or unilateral hearing loss are at greater development risk (43) than their peers without hearing loss. The lower scores and large variation in scores on the caregiver-reported psychosocial HRQoL domain, as compared to the other HRQoL domains, may be a demonstration of this developmental risk. Such a result aligns with other reports of poorer quality of life in school and social domains for children with unilateral hearing loss (44). Uncertainty on appropriate management of hearing loss may also lead to this perception of development risk, with caregivers and audiologists recently reporting challenges in decision-making around best ways to support children born with mild bilateral hearing loss (16, 17).

#### 4.4.2. Unilateral auditory neuropathy spectrum disorder

Our finding that, on average, children with unilateral ANSD demonstrated language performance similar to those with unilateral sensorineural profound losses is novel but not unexpected when considering what is known of outcomes for children with bilateral ANSD. Children with bilateral ANSD, typically in an early-identified setting and users of amplification (hearing aids or cochlear implants), have been shown to demonstrate early language abilities (up to 7 years of age) not significantly different to their peers with bilateral sensorineural loss (45, 46). In comparison, very little is documented on the outcomes of children with unilateral ANSD, likely a consequence of the rarity of this type of unilateral hearing loss (47). By including children with unilateral ANSD in our descriptive study, we are able to report on language outcomes in this under-described group.

## 5. Conclusions

Routine early identification of mild bilateral and unilateral hearing loss has driven recent focus on understanding outcomes for impacted children and their families. Whilst population-based studies, such as this one, describe the unadjusted development outcomes under contemporary detection methods, understanding the factors that mediate these outcomes is required to guide what steps will optimize appropriate support for these children. In particular, more attention needs to be paid in evaluating whether these children have access to or are adequately supported to enroll in early intervention programs, and whether early intervention programs for these children are effective. Similarly, future research needs to focus on the impact of early amplification as well as consistency in amplification use in these children on their language and quality of life outcomes. Efforts to harmonize outcome measures across databanks and projects focused on mild bilateral and unilateral hearing loss, such as the upcoming Australian National Child Hearing health Outcomes Registry (ANCHOR, NHMRC grant 2015735) should enable this transition from descriptive reports to more predictive analyses, particularly as the number of early detected children with these degrees of loss continues to grow.

## Data Availability

The raw data supporting the conclusions of this article will be made available by the authors, without undue reservation.
