# Supplementary material for: Language and health-related quality of life outcomes of children early-detected with unilateral and mild bilateral hearing loss
Source: Front Pediatr. 2023 Aug 14;11:1210282. doi: 10.3389/fped.2023.1210282 (PMC10461396; doi:10.3389/fped.2023.1210282)
Supplement: Supplementary file 1 [file Table1.docx]

**Supplementary Table 1.** Characteristics of non-responders across the three study samples.

|  |  | | | **Age 2 years** | |  | **Age 5-7 years** | |  | **Age 9-12 years** | |
| --- | --- | --- | --- | --- | --- | --- | --- | --- | --- | --- | --- |
|  |  | | | n=86 | |  | n=48 | |  | n=10 | |
|  |  | | | Missing  n (%) |  |  | Missing  n (%) |  |  | Missing  n (%) |  |
| **Hearing-related characteristics** | | | |  |  |  |  |  |  |  |  |
|  | Age at detection/diagnosis (months) – median (IQR) | | | 7 (8.14) | 1.20 (1.20, 1.20) |  | 3 (6.25) | 1.20 (1.20, 2.40) |  | 3 (30.00) | 1.82 (2.62, 2.28) |
|  | Hearing loss severity – n (%) | | | 0 (0) |  |  | 0 (0) |  |  | 0 (0) |  |
|  |  | Bilateral: | Mild |  | 34 (39.53) |  |  | 23 (47.92) |  |  | 4 (40.00) |
|  |  | Unilateral: | Mild |  | 10 (11.63) |  |  | 3 (6.25) |  |  | 2 (20.00) |
|  |  |  | Moderate |  | 13 (15.12) |  |  | 3 (6.25) |  |  | 2 (20.00) |
|  |  |  | Severe |  | 12 (13.95) |  |  | 8 (16.67) |  |  | 2 (20.00) |
|  |  |  | Profound |  | 9 (10.47) |  |  | 7 (14.58) |  |  | 0 (0) |
|  |  | ANSD (unilateral) | |  | 8 (9.30) |  |  | 4 (8.33) |  |  | 0 (0) |
|  | Type of hearing loss – n (%) | | | 0 (0) |  |  | 0 (0) |  |  | 0 (0) |  |
|  |  | SNHL | |  | 71 (82.56) |  |  | 41 (85.42) |  |  | 8 (80.00) |
|  |  | Auditory Neuropathy | |  | 8 (9.30) |  |  | 4 (8.33) |  |  | 0 |
|  |  | Mixed HL | |  | 5 (5.81) |  |  | 1 (2.08) |  |  | 0 |
|  |  | Conductive HL | |  | 2 (2.33) |  |  | 2 (4.17) |  |  | 2 (20.00) |
|  |  | Not available/applicable | |  | 0 (0) |  |  | 0 (0) |  |  | 0 |
| **Demographic characteristics** | | | |  |  |  |  |  |  |  |  |
|  | Sex of child: Female – n (%) | | | 0 (0) | 44 (51.16) |  | 0 (0) | 21 (43.75) |  | 0 (0) | 2 (20.00) |
|  | Socioeconomic disadvantage (SEIFA) – mean (SD) | | | 0 (0) | 1003.89 (74.92) |  | 0 (0) | 1008.00 (59.83) |  | 0 (0) | 960.90 (60.52) |
|  | Household income – n (%) | | | 7 (8.14) |  |  | 11 (22.92) |  |  | 1 (10.00) |  |
|  |  | < $31,199 | |  | 7 (8.86) |  |  | 4 (10.82) |  |  | 0 (0) |
|  |  | $31,199 - $51,999 | |  | 14 (17.72) |  |  | 6 (16.22) |  |  | 1 (11.11) |
|  |  | $52,000 - $103,999 | |  | 35 (44.30) |  |  | 11 (29.73) |  |  | 7 (77.78) |
|  |  | > $104,000 | |  | 23 (29.11) |  |  | 16 (43.23) |  |  | 1 (11.11) |
|  | Household primary language – n (%) | | | 22 (25.58) |  |  | 18 (37.50) |  |  | 10 (100) |  |
|  |  | English only | |  | 34 (53.13) |  |  | 19 (63.33) |  |  | — |
|  |  | Bilingual/multilingual (English + other) | |  | 19 (29.69) |  |  | 8 (26.67) |  |  | — |
|  |  | Other language(s) only | |  | 11 (17.19) |  |  | 3 (10.00) |  |  | — |
|  | Maternal education – n (%) | | | 16 (18.60) |  |  | 14 (29.17) |  |  | 7 (30.00) |  |
|  |  | Year 10 or less | |  | 13 (18.57) |  |  | 3 (8.82) |  |  | 1 (33.33) |
|  |  | Year 11 | |  | 4 (5.71) |  |  | 2 (5.88) |  |  | 0 (0) |
|  |  | Year 12 | |  | 25 (35.71) |  |  | 10 (29.41) |  |  | 0 (0) |
|  |  | Tertiary or postgraduate | |  | 28 (40.00) |  |  | 19 (55.88) |  |  | 2 (66.67) |
| **Health-related characteristics** | | | |  |  |  |  |  |  |  |  |
|  | NICU admissions: yes – n (%) | | | 2 (2.33) | 16 (19.05) |  | 6 (12.50) | 3 (7.14) |  | 1 (10.00) | 2 (22.22) |
|  | Gestational age – mean (SD) | | | 0 (0) | 38.63 (1.96) |  | 1 (2.1) | 38.54 (2.24) |  | 0 (0) | 37.43 (2.11) |
